# “Living Between Two Different Worlds”: Experiences of Leaving a High-Cost Religious Group

**DOI:** 10.1007/s10943-021-01397-1

**Published:** 2021-08-17

**Authors:** Maria Björkmark, Peter Nynäs, Camilla Koskinen

**Affiliations:** 1 Department of Caring Science, Åbo Akademi University, Vaasa, Finland; 2 Centria University of Applied Sciences, Talonpojankatu 2, 67100 Kokkola, Finland; 3 Faculty of Arts, Psychology and Theology, Åbo Akademi University, Turku, Finland; 4 Department of Caring and Ethics, University of Stavanger, Stavanger, Norway

**Keywords:** Religious disaffiliation, High-cost religious group, Experiences, In-depth interviews, Thematic analysis

## Abstract

The aim of this interdisciplinary study is to gain a comprehensive understanding of individuals’ subjective experiences after leaving a high-cost religious group and how these experiences have affected their lives. In-depth interviews were done with 18 participants who had left different religious communities in Finland. The interviews were analysed through a thematic analysis. The results show that religious disaffiliation is a life change that may affect an individual’s life in profound ways. Life after being a member of a high-cost group may involve experiences of fear, guilt, sorrow, pain, loss and even suffering on an existential level. These experiences can have serious implications for one’s well-being and health. However, life after religious disaffiliation also includes many positive aspects, such as experiences of joy, freedom, relief, gratitude and empowerment.

## Introduction and Aim

Religious disaffiliation has become more common in recent years (Fenelon & Danielsen, 2016; Fisher, 2017). Finland is considered a secular country, and secularization is increasing in terms of decreasing memberships and participations in religious services. Even if there is an exceptionally high rate of membership in the state church, the Evangelical Lutheran Church of Finland, this membership is constantly declining, from 95% in 1950 to only 68.6% in 2020. At the same time, religious diversity is increasing, and there are a growing number of new religions and religious movements in Finland (Evangelical Lutheran Church of Finland, 2021; Lassander & Nynäs, 2016; Nynäs et al., 2015). There is no reason to believe that the trend of leaving religion always results in difficulties for an individual. However, in this study, we explore the circumstances where religious disaffiliation can be expected to lead to challenges in an individual’s life.

Researchers have previously found faith and religion to be positive resources that strengthen an individual’s quality of life (Griffith, 2010; Hintikka et al., 2001; Koenig, 2009). Faith can give an individual well-being in the form of hope and joy as well as aims and meaning in life (Koenig et al., 2012), and religion can provide believers with a positive worldview, which gives meaning even to negative experiences (Pargament et al., 2000). Religious affiliation serves as a considerable source of social support for many as social relationships and social integration are important. However, research shows that religion and group membership can also be a burden and negatively affect an individual’s health and well-being. According to Heino (1995), it depends on the character, aim and approaches of a religious community if the community constitutes a resource or a threat for the individual, if it promotes or inhibits health.

Leaving a religious community or one’s faith has been conceptualized with a variety of terms including *dropping out, exiting, defecting, apostasy, disaffiliation and disengagement* (Bromley, 1988). The term *deconversion*, leaving a religion, as a contrast to the term conversion, is also commonly used (Streib, 2021). Studies on conversion have been conducted for decades (Rambo, 1999; Taylor, 2021), but according to Gooren (2007) this research lacks attention to disaffiliation. According to Streib and Keller (2004), the process of deconversion involves intellectual doubt, emotional uneasiness or distress, and moral criticism culminating in disaffiliation from a religious organization. Deconversion is a multidimensional process that leads to changes in key constructs such as values, well-being and spiritual self-identification (Streib, 2021).

The concept religious disaffiliation was chosen in this study because the focus is on individuals leaving a religious group, not on whether they also leave their faith or religion. Religious disaffiliation is most commonly defined as changes in either “Individual role-related activity”, no longer being a member of or involved in the activities of an organization, or “Individual symbolic connectedness”, no longer identifying oneself with a specific religious group or its belief system (Bromley, 1991, p. 165). Bromley (1988, 1991) was one of the first to study religious disaffiliation and the challenges that individuals experience. These difficulties depend mostly on the religious group, how much individuals have invested in the group and how long they have been members (Bromley, 1991). In this study, we explore individuals’ subjective experiences of leaving a religious affiliation in a Finnish context.

Not all who leave a religious community experience hardships and serious implications. Therefore, the participants in our study have been members of what they experienced as being so-called high-cost religious groups, because that is where the challenges seem to be the most visible. High-cost groups are defined as “the most demanding, high-cost, theologically and culturally exclusive religious groups” (Scheitle & Adamczyk, 2010, p. 326). In these groups, members face high levels of participation as well as restrictions on behaviour and social interactions. The Church of Jesus Christ of Latter-day Saints (commonly called Mormons) and Jehovah’s Witnesses are typically considered being among such high-cost groups (Scheitle & Adamczyk, 2010). High-cost groups are described as environments with a strong group dimension, where the group controls its members’ relationships, both with each other and with outsiders. Membership is inseparably connected to an individual’s identity, so an individual considering leaving the group is faced with a difficult choice, because leaving the group entails much more than just leaving an organization. Studies show that leaving these high-cost groups can lead to poor health both physically and psychologically (Coates, 2010; Ransom et al., 2021; Scharp & Beck, 2017).

Previous studies show that leaving a religious group can be experienced as difficult, mostly due to the resulting losses, including that of family and community (Coates, 2010). Religious disaffiliation can significantly impact family systems and lead to the loss and diminishment of social relationships and social support (Knight et al., 2019) as well as experiences of ostracism and social shunning (Ransom et al., 2021). Disaffiliates may have difficulties fitting into society and can feel “out of place” (Coates, 2010) as well as needing to develop a new identity and new self-concepts after leaving the group (Nica, 2020). Life after leaving a religious community can even entail suffering of life, such as living with sorrow, fear, guilt and shame, which can be difficult to endure (Björkmark et al., 2021).

Many popular science and biographical books have been written during the past years, describing religious disaffiliation and related topics, including spiritual abuse, fear and children in controversial groups (Dam, 2017; Egedius & Torp, 2017; Essen, 2008; Finch, 2019; Frisk et al., 2018; Hassan, 2013; Hurtig, 2013; Järvå, 2009, 2021; Linjakumpu, 2015; Linjama, 2014; Rova, 2016; Ruoho, 2017; Villa, 2013). Recently, the topic has also been presented in a handbook from a variety of perspectives (Enstedt et al., 2020). However, few studies have comprehensively explored individuals’ experiences of how disaffiliation affects their lives (Fenelon & Danielsen, 2016; Fisher, 2017), and this motivates the present study as well as further research.

Religious disaffiliation may lead to such profound changes in life that it affects individuals’ health and well-being, and they need to seek help from health care professionals. On the other hand, individuals have their own resources, which promote health and give them strength in such a situation (Nica, 2019; Ronimus, 2011). Religious disaffiliation has been studied from the perspectives of sociology, theology, psychology and the psychology of religion. However, there is a lack of studies in this context from a caring science perspective. In comparison with other disciplines caring science can pay specific attention to an individual’s well-being, health and positive resources.

The aim of this study is to gain a comprehensive understanding of individuals’ subjective experiences after leaving a high-cost religious group and how these experiences have affected their lives.

The study is thus explorative, which is a methodological approach specifically suitable for research where a given phenomenon is not clearly defined and there is only little systematic knowledge about it. This broad-ranging and purposive qualitative methodological approach leads to a description and an understanding of a given topic. Exploration is primarily carried out through inductive methods of research with the goal of generating new ideas and forming theory that emerges directly from data (Stebbins, 2001). Consequently, the study is not driven by a specific hypothesis, even though it is informed by theoretical observations. The study is not comparative in regard to how or when the disaffiliation took place or in regard to experiences of mental health problems and emotional experiences before and after the disaffiliation. Nor is the aim of the study to examine the background to or reasons why the participants disaffiliated.

## Interdisciplinary Theoretical Framework

This study is interdisciplinary and combines the perspectives of both caring science and the study of religions, as they converge and complement each other. The study’s theoretical framework consists of a theory about communion from a caring science perspective and of social identity theory from a study of religions perspective. These theories can shed light on and promote understanding of individuals’ experiences after religious disaffiliation.

The study is grounded in caring science, a human science that adds to the understanding of the human being, with the ultimate goal of alleviating suffering and serving life and health (Eriksson, 2006). A central concept in caring science (Eriksson, 2006; Lindström et al., 2018) is communion, which is used as a positive qualitative aspect of belonging to a community. Belonging to a group is considered one of the basic needs of a human being, and fellowship, or communion, is important for health and well-being. Human beings long to be unique, and simultaneously to be part of a larger communion, to exist as a person in a context and find meaning in life through communion. A caring communion is a place where a person feels invited, welcome and can experience love, joy and freedom. To not feel welcome deprives a person of hope and joy in life, regardless if in a single, concrete situation or life as a whole.

Social identity theory has also been chosen as a theoretical framework as it serves as a structure for understanding the individual as member of a group. Social groups are the most important expression of humans as social beings with a need to live in social groups. The social identity theory was developed by Tajfel and Turner in the 1970s. Groups provide us with a sense of *social identity*, which is defined as the knowledge that we belong to certain social groups together with the emotional and value significance that this group membership brings us. Groups form an external framework for our behaviour, and they also shape our psychology and contribute to our sense of self. An individual’s sense of self is enhanced not only by belonging to certain groups, but also by being different from members of other groups. These distinctions between “us” versus “them” help us understand ourselves and also affect our self-evaluation and sense of worth (Haslam et al., 2009).

## Method, Data and Analysis

Recruitment of participants was carried out among persons who had left a religious community and had experienced difficulties due to this disaffiliation. Recruitment was mainly done through the organization Support for Victims of Religions (2021), by posting research requests on their web-pages and in closed Facebook peer support groups. This is a national organization in Finland, whose goal is to support and give peer support to people who have experienced difficulties in religious communities. Inclusion criteria involved participants who had left groups, which they themselves perceived as being high-cost religious groups, as well as groups that have been established in Finland over a longer period of time.

In-depth interviews were conducted with 18 participants who had left different religious communities in Finland. The participants are presented in Table 1. The information gathered about the participants was gender, age, place of residence, religious community they disaffiliated from and years since disaffiliation. Other information, such as education and mental and physical health status, was deemed as not relevant for this study. As Finland is a small country, the participant’s anonymity has to be protected and all of their information cannot be disclosed.

**Table 1:** Participants

Former religious community disaffiliation	Gender	Age	Years since
Free Church	Man	25–30	11
Jehovah’s Witnesses	Woman	45–50	3
Jehovah’s Witnesses	Woman	55–60	8
Jehovah’s Witnesses	Woman	40–45	19
Jehovah’s Witnesses	Woman	30–35	16
Jehovah’s Witnesses	Man	30–35	9
Jehovah’s Witnesses	Woman	45–50	16
Jehovah’s Witnesses	Man	40–45	15
Laestadianism	Woman	35–40	5
Laestadianism	Woman	40–45	4
Laestadianism	Man	45–50	1
Laestadianism	Woman	60–65	35
Laestadianism	Woman	50–55	7
New charismatic movement	Woman	35–40	3
New charismatic movement	Woman	35–40	3
New charismatic movement	Woman	40–45	7
Pentecostalism	Woman	50–55	9
Pentecostalism	Man	45–50	13

Participants’ information is presented in a form that protects their anonymity

The majority of the participants (14) had been members of the religious community since birth, while 4 had affiliated as adults. Also, 14 participants had disaffiliated voluntarily, while 4 had been excommunicated by the community (involuntarily). The religious communities that the participants had left were: Jehovah’s Witnesses, Laestadianism (a pietist revival movement, part of the ELCF church), New charismatic movements, Pentecostal congregations and one Free Church (part of the Evangelical Free Church of Finland). All of these communities can be considered high-cost religious groups.

The in-depth interviews were conducted by the first researcher. A semi-structured interview guide was made in advance, including questions about life after the disaffiliation, how their health and well-being had been affected, and what they had experienced as particularly positive and especially difficult after the disaffiliation. The interview began by giving information about the study, and written consent was obtained from all participants. The interviews lasted from 1 to 2 h with each participant and were mostly conducted in person; however, two interviews were conducted via Skype, according to the wish of the participants. After the interview, the participants were asked about their experiences of it, and if needed, information was given about where to find support (peer support and helplines). The interviews were recorded (a total of 26.08 h) and transcribed, and the data material consists of 328 pages transcribed text (Times New Roman, 12 p, single space). Anonymization was done through numbering the interviews, and only the first researcher knows the identity of the participants.

The thematic analysis (TA) method by Braun and Clarke (2006) was chosen. TA is “a method for identifying, analysing, and interpreting patterns of meaning (themes) within qualitative data” (Clarke & Braun, 2017, p. 297). An inductive analysis was conducted. This is a data-driven analysis, where the themes are strongly linked to the data without fitting them into a pre-existing theoretical frame (Braun & Clarke, 2006). The phases of the TA process were followed: familiarization with the data, generating initial codes, searching for themes, reviewing themes, defining and naming 20 sub- and five main themes and finally producing the report (Braun & Clarke, 2006). The coding phase was done with the help of NVivo, a computer-assisted qualitative data analysis software (CAQDAS).

### Ethical Considerations

Ethical aspects become especially important as this study can be considered “sensitive research” with vulnerable participants (Liamputtong, 2007). An ethical approval was received from the Board for Research Ethics at Åbo Akademi University on 30 May 2018. The study has been carried out in accordance with the ethical principles of Research Integrity in Finland (Finnish Advisory Board on Research Integrity, 2012; Finnish National Board on Research Integrity, 2019). It has been crucial through the entire research process to be attentive to the fact that religious disaffiliation may be a sensitive and complex subject, which may bring difficult memories and emotions to the surface. Permission was obtained to recruit participants in Facebook groups and on web-pages, and contacts were created in a tactful and dignified manner. It was important to not harm the participants or cause unnecessary difficulties. All participants were informed about the purpose and procedure of the study, and participation was voluntary and could be interrupted at any time. Special attention was paid to protecting the informants’ integrity and anonymity throughout the entire study.

## Results

Five themes were found through the qualitative thematic analysis: living with fear and guilt, sorrow and pain over what one has lost, broken as a human being, lifelong process of building a new identity, and a life of freedom and joy. The themes and subthemes are presented in Table 2. The reliability of the themes is confirmed by quotes, and numbers after the quotes refer to which interview the quote is taken from.

**Table 2:** Themes and subthemes

Theme	Subtheme
Living with fear and guilt	Living with different kinds of fears Fear of rejection and difficulties trusting others Deep feelings of guilt Feelings of self-blame and shame
Sorrow and pain over what one has lost	Losing family and friends Rejection, loneliness and isolation Void and emptiness in life Sorrow over the sacrifices made
Broken as a human being	Mental health problems and mental illness Psycho-somatic symptoms and physical illness Experiences of trauma and violence Substance abuse and suicidal thoughts
Lifelong process of building a new identity	Living between two different worlds Identity confusion and identity crises Finding one’s true self and new ways of looking at the world Building a new life is a lifelong process
A life of freedom and joy	Experiences of joy, happiness and well-being Living with a sense of freedom and relief Feelings of empowerment, strength and courage A new world opened up

### Living with Fear and Guilt

The participants described how they had lived all their lives with different kinds of fears, and these fears continued, even after disaffiliation. One participant used the metaphor of having been marinated in shame and fear, all through her childhood. She said: *When you have been marinated in it as a child… in shame and fear… You can’t get rid of it…. you have to learn to live with it… (18).*

The participants expressed how they not only had to deal with fears related to this present life, but also fears related to death and eternity, such as fears that they had sinned, fear of punishment and fear of going to hell. *Overall, I think this is such a difficult topic… because it is not only about things of this life, but there is this eternity perspective… that if I leave this congregation… and end up in hell for eternity… then how do I dare leave…if this is the alternative… (3).*

Freedom was described as wonderful, but in the beginning, also as frightening. The participants described being afraid of having gone astray and doing wrong. Also fears related to other people, including fear of rejection and being afraid of losing people close to them. This led to having difficulties trusting anyone or anything. One participant said: *I don’t let people come close… I believe it is because some kind of fear… fear of losing them… (14).*

Religious disaffiliation also leads to deep feelings of guilt. Having lived many years with the feeling of being a sinner and an unworthy person, it is difficult to get rid of the guilt. Guilt over being the one who is wrong, and that the others were right after all, or feeling a sense of being punished by God with illness or other difficulties. Some described that their earlier conception of God was that God was the same as this group, and now they felt as they were eternally lost. The participants who had joined the community as adults felt guilty over having joined and that they now deserved the hardships. *I still have those guilty thoughts. Or I think that if things go a certain way it is because God is punishing me… Or that they are right and I am lost and wrong… (4).*

Self-blame and shame are connected to feelings of guilt, blaming oneself for joining the community, for wasting so many years there, and for the feeling of being manipulated. Feeling shame over what one’s children have had to endure and wanting to honour one’s parents, but now causing them pain. Feelings of having been manipulated and cheated can lead to anger and bitterness. *Something that has bothered me many times since I left, is that I wasted so many years…and I blame myself… I think about it often…that why did I stay there so long, why did I give my whole youth and all my years away…and why did I do as I was told… this is really difficult for me from time to time. (10).*

### Sorrow and Pain Over What One Has Lost

Religious disaffiliation can lead to great changes and losses in life. For some it may entail losing one’s whole family and all friends at once. *All my friends were in that congregation, and at the point when I was excommunicated, I lost everything, absolutely everything… (11). All the people around you disappear and all your friends leave….and all the familiar, safe things in life disappear, all at the same time. (7).*

Some participants described a total rejection and a sense of “ceasing to exist” in the eyes of those who are still members, as their former community practices official exclusion. Others described more unofficial and unspoken forms of exclusion. All participants spoke about condemnation and feelings of being an outcast. *How total the loss is… when you practically lose all the people around you… who you have been in contact with on a daily basis…you don’t have anyone you can call…. (11).*

Sorrow comes from missing one’s children, parents and friends. Pain also comes from knowing that one’s choices have caused one’s own family to suffer. Some experienced a lost youth, and having to relive that now as an adult. *Mother’s Day is a day, when I see that my adult friends are for instance with their parents or doing things with their mom…or if I see someone travelling with their mom…or just spending time with their mom…that’s when I feel sorrow… because I lost that… I have been really close to my mom…. we had a real close relationship… (14).*

Some participants had suffered from great loneliness and social isolation. Others missed the unique fellowship of the community and felt an emptiness and a spiritual longing. Disappointment arises from understanding that love, respect, acceptance and support, within the community, were conditional. Feeling disappointed with former friends and also with leaders in the community. In the long run, all participants had to sacrifice something, some more and some less, in order to receive freedom and a good life.

### Broken as a Human Being

The participants described their experiences of mental health problems, or being “broken and cracked as a human being”. Almost all participants spoke about feelings of anxiety and they also mentioned stress, fatigue, burnout, panic attacks and mood swings. Psycho-somatic symptoms, such as sleep disturbances, digestive problems and headaches, were common and associated by the participants with their disaffiliation. Some even described how the stress after disaffiliation triggered physical illnesses that in some cases became chronic. *Chronic stomach problems have remained since then, I have had lots of stomach and bowel problems. Migraines were activated around the same time… they were really regular and chronic. I believe they were connected… things were so difficult at the time… (12).*

Right after the disaffiliation there were experiences of emotional numbness, of feeling empty and incapable of showing or expressing feelings. Some mentioned that they had tried to suppress their pain with alcohol and ended up drinking excessively, for a period of time. *There the gap between me and the congregation started …and it grew… and I started using more alcohol… (15).*

According to the participants, some of their mental health problems were induced by fear. Some experienced fear and nightmares about perishing and the afterlife, such as fear of Harmageddon and hell. Some experienced frightening thoughts and voices, while others experienced social fears, which lead to social anxiety and isolation from others. *I had tons of anxiety… it felt like this town is such a small place, I can’t go anywhere… (4).*

Several participants spoke about feelings of being traumatized inside the community. Some were victims of abuse, as they had experienced violence during childhood, and others had witnessed sexual abuse. *I started having very violent flashbacks from my childhood…. I come from a violent family….and suddenly this started showing up, as very strong flashbacks (8).*

Some were diagnosed with mental illnesses, such as severe depression, anxiety disorders and psychosis, which required sick leaves of different lengths, and even in-patient psychiatric care. *I suffered from severe depression…. It was life threatening; I was dangerous to myself and to those around me… (13). I had strong anxiety… and before the speed and mania turned to psychosis… I had very strong anxiety that then triggered the psychosis… (1).*

Some participants had gone through periods in life when their pain was bottomless, when they felt fragile and were not able to cope with life. Some had suicidal thoughts, since death felt easier than living with the anxiety and pain. *At that point it would have been easier to commit suicide… than living with the agony….it would have been possible if I would have had an appropriate way to do it… (6). I totally lost my sense of security, when I hit rock bottom…. I was so full of anxiety and pain, that death would have been easier than the anxiety and pain…. (6).*

### Lifelong Process of Building a New Identity

Life after religious disaffiliation is described as living between two different worlds. A strong border between one’s own community and the outside world, and also the distinction between “us and they” create a sense of two different worlds. In the beginning, there were feelings of “floating in emptiness” and feelings of outsidership, feeling that one does not belong to either world. Many participants described a complete lack of knowledge about the outside world. Building a new life involved learning many new things about this world. Some described how everything had to be relearned and built from the ground up. *I have a feeling that my whole identity was founded on that (religion), and at that point when I left…or I was in the process of leaving… I realized that I didn’t know anything about this world. (7) Like you are an immigrant and have to abandon all your ways of doing things and learn a new way of living in this society. (8).*

The participants described that their former identity had largely been connected to the community identity. Life in the community was about life as a whole, not just one small part. After disaffiliation, they experienced feelings of identity confusion and identity crises. Since their personal and community identities were so intertwined, in the beginning it was difficult to distinguish one’s own identity. Some participants described coming out as a homosexual, which led to even greater challenges in life.

Part of building a new identity as a disaffiliate is described as finding one’s own true self, finding own ways of thinking and listening to own wishes and choices. One’s former life, within the community, was experienced as easier in a way, because there one was told how to think and how to act. Making own choices and starting to think for oneself was at first both liberating and difficult. Eventually one needs to create own opinions and new ways of looking at the world. *When you seriously start thinking about your own identity at 50…what am I, who am I, what do I believe in, do I approve of this, do I approve of that…. what is my opinion. (7).*

To become free from a previous identity means that in the beginning one’s direction in life may be completely lost and beginning to look at the world in a completely new way is a long process. While the physical level of disaffiliation (ending physical attendance) can be a short process, separation on mental, emotional and spiritual levels is described as a very long process. One participant described how she had left the community 35 years ago, and still the process was on-going. *Breaking free on a physical level can be quite a quick process… but breaking free mentally can last many years… (15).*

The participants described how building a new identity is a lifelong process and how their background might affect them for the rest of their lives. Some described how their background had left them with such emotional scars that they doubted if they ever would find balance in life. Others experienced that they already had reached a point of a balanced and good life. *Maybe on the same level as when you say that “once an alcoholic—always an alcoholic” … I believe that when you have been under strong mind control since childhood, it is the same way…I can’t even say that it is a part of me, but that I am still a part of it in some way. You could even say that you need treatment all your life, at least at some level. (1).*

### A Life of Freedom and Joy

The participants also described many positive aspects of disaffiliation such as joy, happiness and a sense of freedom. The decision to leave is described as one of the biggest and most satisfying decisions in life. *I felt free, like being cut loose from a ball and chain… It was an amazing feeling (5). It was the greatest and most satisfying thing… when I left everything behind… I was so happy… it just felt so unreal… I felt like I was floating several feet up in the air, all the time… (13).*

Freedom is partly about feeling a sense of relief over having left that chapter of life behind, and not having to go back. Some even described it as getting out of prison. Freedom is also about finally being able to be oneself, and be able to live a life one never has dared dream of.

*I remember those first feelings of freedom…when I suddenly realized that I am no longer tied down by chains in any direction. I can do whatever I want and listen to whatever I want… (2).*

The decision to disaffiliate required courage and strength. Some participants described there being a certain “turning point” that had led to their decision, but most described their disaffiliation as a result of a very long process. Many came to a point where there were no other alternatives, and they felt that their decision was certain and final. *I knew that the day I dropped.*

*out….no one could persuade me to come back…I was absolutely sure. (18) I didn’t leave until I had tried everything… when I didn’t have anything left… Then I was totally sure about my decision. (10)*

The decision to leave gave feelings of strength, security and courage, as well as an empowering feeling of having dared to leave and being able to take responsibility over one’s own life. The decision was expressed as being a difficult one, but liberating at the same time. *The decision…when I decided to take responsibility for my own life and do things that are important to me, that was so empowering….it gave me a sense of security, that I had a possibility to try and do my own things. (15).*

The positive experiences and feelings came with a price and were mixed with negative feelings and difficult experiences. Life, at least in the beginning, was described as a movement back and forth between well-being and difficulties, for instance, as going back and forth between feelings of freedom and fear. Yet, the participants expressed a deep gratitude and appreciation for their new life and newfound freedom. They experienced that a new world had opened up, a new world with novel possibilities.

## Discussion

The results of our study show that leaving a high-cost religious group is described as an experience of living between two different worlds. At first, the participants describe themselves as being outsiders in a new world and feeling like living in an “in-between” space, not belonging anywhere. This corresponds to the results of Bromley (1991, p. 175) and Ebaugh (1988, p. 113) who describe how members who have been deeply involved in religious groups experience feelings of “having their feet in two worlds” and being “caught between two worlds”. As our study shows, participants felt like they were immigrants who knew nothing about the new culture and had to learn everything from the beginning. Gradually, they found more and more elements of the new world that helped them in building a new life with new content.

The results of our study correspond with previous research that suggests that religious disaffiliation may lead to major adjustments in life, be emotionally and relationally challenging and may cause significant disruptions in social relationships. Religious disaffiliation may result in significant changes in a person’s identity and life course and also affect quality of life and well-being (Fenelon & Danielsen, 2016; Knight et al., 2019; Scheitle & Adamczyk, 2010), which our study also shows. Our results suggest, in accordance with Bromley (1991), that the severity of the disaffiliation process seems to depend on factors such as duration of membership, group involvement and manner of leaving the group. However, as we have not conducted a comparative study, this is an area that still needs to be explored.

The results can be understood in the light of social identity theory and how social groups contribute to a sense of social identity. The participants put into words how they had lost their social context, and through this also their social identity. Especially if one has been born into a group, and one’s social identity is entirely connected to being a member of this group, losing this identity and rebuilding a new identity can be a very long process. Loss of social identity is known and is also shown in this study, to cause health problems, namely mental health problems. In our study, we found that all participants experienced a social identity crisis, caused by doubt and negative experiences in the group. Extensive emotional effort is required in order to create a new social identity.

In our study, we also found how important communion, fellowship or social context is in a person’s life. Most participants described how they had been members of communions, which they once experienced as loving and caring and where they had been invited and welcome. For various reasons, that were not explored in this study, the participants had gradually begun to experience that the communion no longer held the same attributes that it once had held for them. Many described how there was a growing conflict between striving to be a unique person, on the one hand, and measuring up to the demands and the teachings of the group, on the other. The previous communion could no longer provide love, joy, freedom or meaning in life. The realization of not being welcome anymore or of being rejected was very painful for the participants. For some, the feeling of not being welcome was limited to certain situations and certain people, while for many this feeling encompassed all areas of life, all relationships and life as a whole. This process was described by many as “losing oneself”, while adjusting to a new kind of life and a new identity.

The results of this study show that experiences differ depending on when a person has joined the religious group and how the disaffiliation occurred. The study reveals a significant difference between disaffiliates who were born into the community and those who affiliated as adults. Disaffiliation for someone who has joined as an adult often means that connections with the “outside world” have remained. What is more, family and friends are significant sources of support after the disaffiliation. On the other hand, a person who has been born into the community may lose all social support as a result of the disaffiliation and end up isolated. Additionally, experiences differ between those who had involuntarily been disaffiliated from the group and those who had made a voluntarily decision. The process of leaving involuntarily may be an unexpected event, and the individual does not go through doubts and negative feelings until after the disaffiliation. Moreover, being officially excommunicated from the group may lead to a deeper suffering than if leaving voluntarily, resulting in, feelings of rejection, guilt and shame.

It is important to emphasize that life after disaffiliation also includes many positive aspects, such as experiences of joy, freedom, relief, gratitude and empowerment. The decision to leave was described as difficult, but as a whole, as a very satisfying decision that brought about freedom and joy. Leaving requires strength and courage, but also gives a person these same positive experiences, feelings of strength and courage. Depending on how much time has passed since the disaffiliation and the kind of process one has to go through, eventually the positive aspects become clearer and more evident in life. All participants drew strength from their own health resources or support that was provided in the process of rebuilding their life. Important in this process was finding new social contexts or new social relationships. Finding new relationships, outside of the previous community, seems to play a vital role in forming a new life, of independence and a new self-understanding.

The results demonstrate that disaffiliation from a high-cost religious group may affect a person’s life in profound ways and may involve suffering, even on an existential level. These experiences can have serious implications for an individual’s well-being and health. The theme being broken as a human being shows how an individual can experience health problems related to both physical and mental health. Even quite serious symptoms of health problems and mental illness were described in this study. The participants expressed that during the process they had needed nursing care, both in-patient and out-patient services. Many had also sought and benefitted from psychotherapy and other forms of therapy.

### Limitations

By describing the methods used in this study, we have strived to maintain credibility. All steps of the analysis process were discussed and reviewed with all co-authors of the study in an attempt to strengthen the reliability and quality of the interpretations. We have presented the findings as themes and confirmed the themes with rich, vivid quotes from the participants. Findings have been described in an honest and careful manner, without shying away from the difficulties that religious disaffiliation entails. The study is empirically grounded and has external value, since the results are significant and can be used in further research.

One challenge in this study has been the translation of quotes, as the interviews were conducted in Finnish and Swedish. We are aware that the original meaning of quotes can be lost in translation, so the translations have been checked by an official translator. We may also have lost some nuances and depth of individual experiences as our priority has been to present the larger variety of themes. That the participants were mainly recruited through the organization support for victims of religions can be considered a limitation in this study, since these persons have sought support because of explicit negative experiences before and after their disaffiliation. However, we chose to recruit persons from this organization, as well as by means of other methods, as our aim was to interview persons who had experienced difficulties due to their disaffiliation. Because of the sampling method and the qualitative and explorative design of the study, generalization is not possible to other groups and contexts.

## Conclusion

Leaving a high-cost religious group may mean significant changes and challenges in one’s life and experiences of living in between two different worlds. At first, one may experience being an outsider in a new world and not belonging anywhere. However, disaffiliation also leads to many beneficial aspects, such as positive feelings, experiences and life changes. Life after leaving a religious affiliation can be described as living in a movement back and forth between difficulties and well-being. Eventually, when one gains one’s “foothold” again, life takes on a new, positive direction.

This research consists of several steps as this first explorative study shows and affirms a need for further studies on many topics relevant for nurses and other health care professionals in regard to religious disaffiliation. Further studies are needed on what kind of coping, care and support a person needs in order to regain balance and health in life. This new knowledge will be important for health care professionals and therapists working with individuals who are experiencing difficulties after religious disaffiliation and is needed to develop the nursing care and support that they need.

## Declarations

### Data Protection, Confidentiality and Privacy

Special attention was paid to protecting the informants’ integrity and anonymity throughout the entire study, as concealing the identity of the participants is essentially important in sensitive research with vulnerable groups. The data are stored according to the data protection regulations at Åbo Akademi University.
